# The Vascular Endothelial Growth Factor Inhibitors Ranibizumab and Aflibercept Markedly Increase Expression of Atherosclerosis-Associated Inflammatory Mediators on Vascular Endothelial Cells

**DOI:** 10.1371/journal.pone.0150688

**Published:** 2016-03-09

**Authors:** Clare Arnott, Gaya Punnia-Moorthy, Joanne Tan, Sara Sadeghipour, Christina Bursill, Sanjay Patel

**Affiliations:** 1 Department of Cardiology, Royal Prince Alfred Hospital, Sydney, New South Wales, Australia; 2 Sydney Medical School, The University of Sydney, New South Wales, Australia; 3 Heart Research Institute, Sydney, New South Wales, Australia; University of Leuven, Rega Institute, BELGIUM

## Abstract

**Introduction:**

Recent studies have suggested that the VEGF inhibitors, Ranibizumab and Aflibercept may be associated with an excess of cardiovascular events, potentially driven by increasing atheroma instability, leading to plaque rupture and clinical events. Inflammation plays a key role in the progression of atherosclerotic plaque and particularly conversion to an unstable phenotype. Here, we sought to assess the *in vitro* effects of these drugs on the expression of key inflammatory mediators on endothelial cells.

**Methods:**

Human coronary artery endothelial cells were co-incubated for 16h with Ranibizumab (0.11nM) or Aflibercept (0.45nM), as determined by each drug’s peak serum concentration (Cmax). Expression at protein (ELISA) and gene (RT-PCR) level of inflammatory chemokines CCL2, CCL5 and CXC3L1 as well as gene expression for the cell adhesion molecules VCAM-1, ICAM-1 and the key NF-κb protein p65 was assessed. VEGF-A protein levels were also determined.

**Results:**

Both drugs significantly increased chemokine, cell adhesion molecule (CAM) and p65 expression, while decreasing VEGF-A protein secretion. At equivalent Cmax concentrations, Aflibercept was significantly more pro-inflammatory than Ranibizumab. Reduction of secreted VEGF-A levels significantly attenuated inflammatory effects of both drugs, whereas blockade of the VEGF-A receptor or silencing of VEGF-A gene synthesis alone had no effect, suggesting that binding of drug to secreted VEGF-A is crucial in promoting inflammation. Finally, blockade of Toll-like receptor 4 significantly reduced inflammatory effects of both drugs.

**Conclusion:**

We demonstrated here, for the first time, that both drugs have potent pro-inflammatory effects, mediated via activation of Toll-like receptor 4 on the endothelial cell surface by drug bound to VEGF-A. Further studies are required to investigate whether these effects are also seen *in vivo*.

## Introduction

Anti-angiogenic therapies, via pharmacological inhibition of Vascular Endothelial Growth Factor A (VEGF-A), have been shown to be highly effective in the treatment of both neoplastic conditions and various eye diseases, including neovascular age-related macular degeneration (a major cause of blindness in the developed world), diabetic macular oedema, retinal vein occlusion and myopic choroidal neovascularisation [1] [2,3] [4] [5] [6]. Currently, the two most widely used VEGF-A inhibitors for vascular eye conditions are ranibizumab and aflibercept, with patients receiving, on average 8–9 intra-ocular doses in the first year of treatment. Ranibizumab (Lucentis; Novartis Pharma AG, Basel, Switzerland, and Genentech Inc., South San Francisco, CA) is a recombinant, humanized monoclonal antibody that binds all isoforms of VEGF-A, developed specifically for intra-vitreal administration [1] [7]. Aflibercept (VEGF-Trap Eye/Eylea; Regeneron, Tarrytown, NY) is a soluble decoy receptor generated with Trap technology, which employs the fusion of components from multiple endogenous receptors. Similar to ranibizumab, aflibercept binds multiple isoforms of VEGF-A, but in contrast it also binds the related VEGFR1 ligands, VEGF-B and PlGF [8] [9] [10] [11] [12].

There is emerging evidence linking intra-vitreal use of ranibizumab and aflibercept with an increase incidence of cardiovascular events, predominantly stroke, particular in those patients already at high baseline risk for cerebrovascular events [13] [14]. Importantly, both drugs have been shown to enter the systemic circulation after intra-ocular injection, though systemic absorption is significantly greater with aflibercept [15] [13] [16]. Such findings are of particular concern as those patients at most risk of developing vascular eye disease and therefore most likely to benefit from intra-vitreal anti-VEGF therapies, often have multiple cardiovascular risk factors, and are therefore also at high risk of future cardiovascular events.

Inflammation plays a critical role in all stages of atherosclerotic plaque and particularly in conversion to an unstable phenotype, leading to the acute coronary and cerebrovascular syndromes [17] [18]. In particular, chemokines, a family of potent chemotactic cytokines, regulate leukocyte trafficking and are abundant at sites of vascular inflammation [19]. Although VEGF inhibitors are known to have a number of cardiovascular side effects—hypertension, proteinuria, hypercoagulability, endothelial dysfunction [20]; their putative-pro inflammatory properties have not yet been explored. Accordingly, we sought to investigate the *in vitro* effects of Ranibizumab and Aflibercept on expression of key mediators, known to participate in atherosclerosis-associated inflammation. We report that both drugs markedly increase gene expression and protein secretion of chemokines on endothelial cells, a process that is dependent on drug binding to secreted VEGF-A with subsequent activation of Toll-like receptor 4. Our findings, therefore, elucidate mechanisms by which these agents may promote atherosclerosis-associated inflammation and subsequent cardiovascular events.

## Methods

### Cell Culture and Incubations

Human coronary artery endothelial cells (HCAECs) (sourced from ATCC) were extracted and cultured by us, used from passage 3 up to passage 6 and maintained in MesoEndo endothelial (Cell Applications Inc, San Diego, CA, USA). To simulate each drug’s systemic Cmax concentration after intra-vitreal injection, ranibizumab and aflibercept were co-incubated with HCAECs for 16h to achieve a final well concentration of 0.11nM and 0.45nM, respectively [15]. Drug treated cells were compared with Phospate-Buffered Saline (PBS) control. For Vascular Endothelial Growth Factor Receptor 2 (VEGFR2) and Toll-like Receptor 4 (TLR4) receptor blocking experiments, HCAECs were simultaneously co-incubated for 16h with ranibizumab or aflibercept and a VEGFR2 blocker (Human VEGFR2/KDR MAb [Clone 89106], R&D Systems [Cat. #: MAB3572]) or IgG Control (R&D systems) before harvesting. Similarly, drug treated cells were simultaneously co-incubated with a TLR4 inhibitor (CAT. # CLI095, Invivogen, San Diego, CA, USA) or Ig control (Invivogen, San Diego, CA, USA) for 16h. Following incubation, cells were washed with PBS and then harvested for subsequent experiments. All experiments were performed in quadruplicate (n = 4) and repeated 3 times.

### SiRNA knockdown

HCAECs were cultured in 6-well plates. Transfection reagent mixture was prepared using Lipofectamine 2000 (Invitrogen) in serum free DMEM and siRNAs to VEGF-A and VEGF-B. (Ambion). HCAECs were then washed with PBS and incubated with the transfection reagent mixture for 5h at 37 C. After incubation, the transfection reagent mixture was replaced by mesoendo media and incubated for a further 48h. Cells were then incubated with ranibizumab (0.11nM), aflibercept (0.45nM) or PBS for 16h before harvesting.

### Cell viability

For all experiments, an MTT assay demonstrated >95% cell viability.

### RNA extraction and quantitative RT-PCR (qPCR)

RNA was extracted using Qiagen RNeasy kit (Qiagen, Valencia, CA, USA) following manufacturer’s instructions. RNA concentration and purity were determined using a Nanodrop Spectrometer 200c (Thermofisher) and only RNA with an absorbance 260/280 ratio ranging from 1.9 to 2.1 was used for experiments. Subsequently, 400ng of RNA was converted to cDNA using the iScript cDNA synthesis kit (biorad, Hercules, CA, USA). QPCR was performed in triplicate on a Biorad CFX96 using 25ng of cDNA template and SYBR green master mix (Biorad) and using primer sequences CCL2: 5’TCATAGCAGCCACCTTCATT-3’, (R) 5’-TCGGAGTTTGGGTTTGCTT-3’, CCL5 (F) 5’-GCAAGTGCTCCAATCTTGCA-3’, (R) 5’-CTTCTCTGGGTTGGCACACA-3’, CX3CL1 (F) 5’-CAGCAGTGACCGGATCATCTC-3’, (R) 5’-TGCTCTGAGGCTTAGCCGTAA-3’, GAPDH (F) 5’-TCCCATCACCATCTTCCA-3’, (R) 5’-AGGCAGGGATGATGTTCT-3, ICAM-1 GGCTGGAGCTGTTTGAGAAC (F), ACTGTGGGGTTCAACCTCTG (R), VCAM-1 CAGACAGGAAGTCCCTGGAA (F), TTCTTGCAGCTTTGTGGATG (R), p65 CCAGACCAACAACAACCCCT (F), TTGGGGGCACGATTGTCAAA (R), VEGF-A (F) CCCACTGAGGAGTCCAACAT, (R) AAATGCTTTCTCCGCTCTGA.

Relative mRNA expression was calculated with the 2 –(Ct) method and normalized to glyceraldehyde 3-phosphate dehydrogenase (GAPDH).

### Enzyme-linked immunosorbent assay (ELISA)

Protein concentrations of MCP1, CCL5 CXC3L1 and VEGF-A in cell supernatants were determined with ELISA (R&D systems, Minneapolis, MN, USA), following the manufacturer’s instructions.

### Statistical analysis

Experimental results were expressed as mean ± standard error of the mean (SEM). The Mann Whitney U test was used to identify differences between experimental groups. Values of P < 0.05 were considered significant.

## Results

### 1. Both drugs stimulate expression of inflammatory mediators in vascular endothelial cells with aflibercept being more pro-inflammatory than ranibizumab

**Fig 1:**
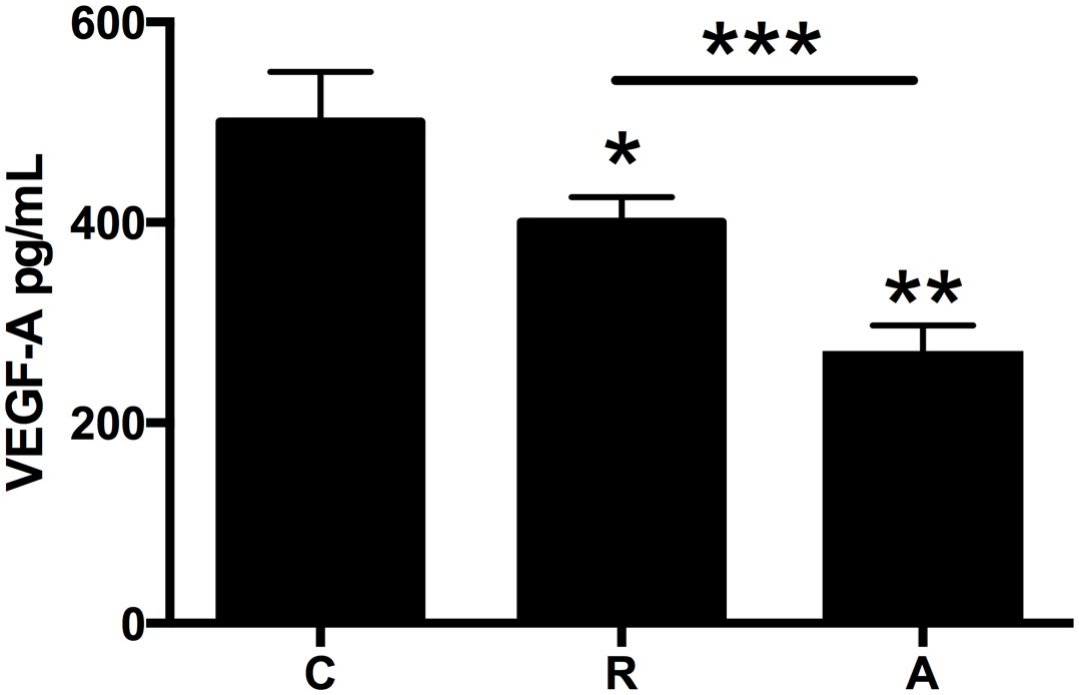
HCAECs were incubated with ranibizumab (R) (0.11nM) or aflibercept (A) (0.45nM), or PBS (C) for 16 h. Changes in VEGF-A protein levels were quantified by ELISA. Results are expressed as means +/- SEM. *P < 0.05 ranibizumab treated cells vs. non-treated cells; **P< 0.05 aflibercept treated cells vs. non-treated cells; ***P<0.05 ranibizumab treated cells vs aflibercept-treated cells.

Culture media were analyzed as described in Materials and Methods for changes in VEGF-A protein levels, chemokine mRNA and protein levels as well as p65 and cell adhesion molecule mRNA levels. Both drugs significantly decreased VEGF-A protein levels (Ranibizumab by 25%, Aflibercept by 45%, p<0.05 for both versus untreated cells, Fig 1).

**Fig 2:**
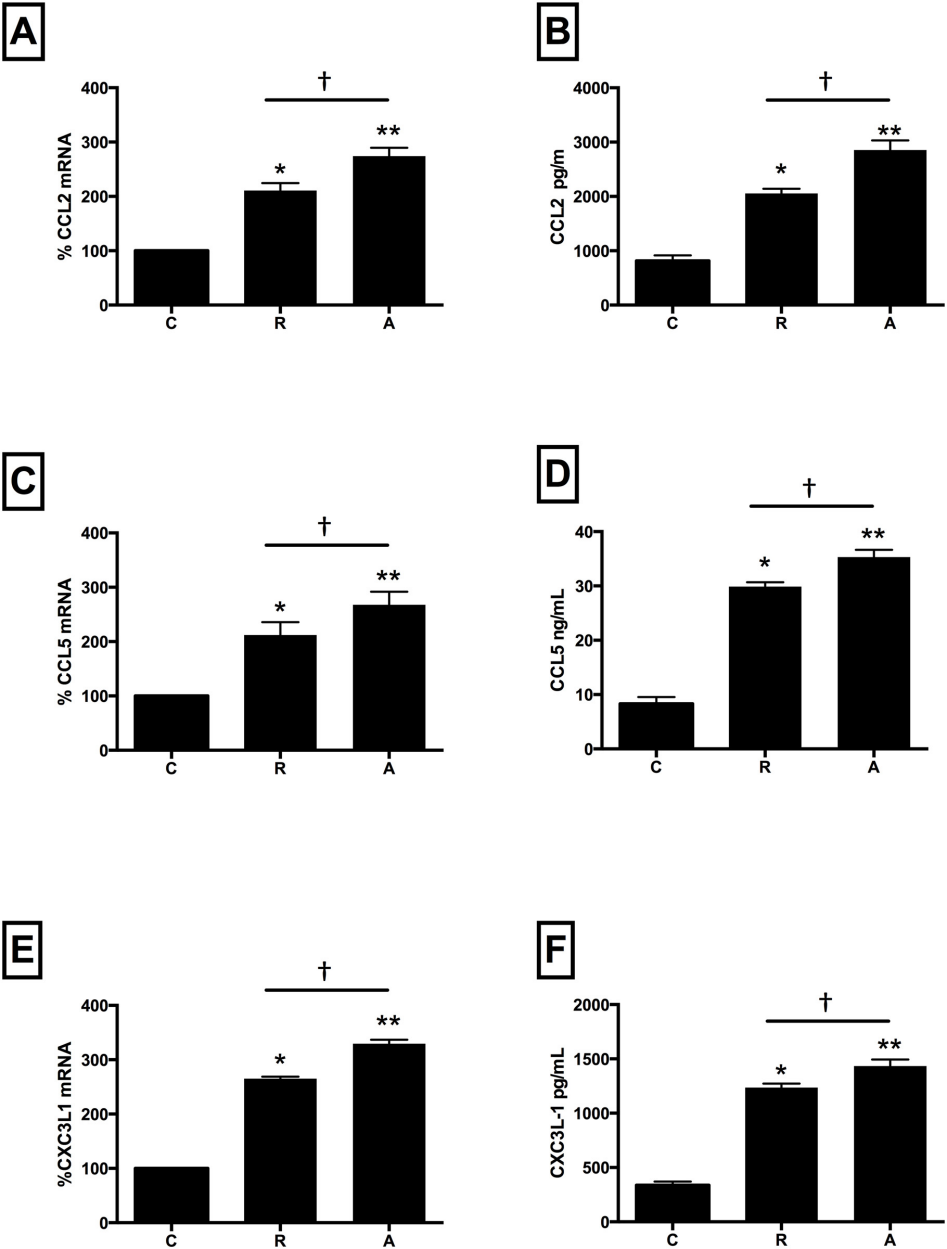
Ranibizumab and aflibercept increase CCL2, CCL5, and CX3CL1 mRNA and protein levels in HCAECs. HCAECs were incubated with ranibizumab (R) (0.11nM) or aflibercept (A) (0.45nM), or PBS (C) for 16 h. Changes in CCL2, CCL-5 and CXC3L1 (panels A, C and E) mRNA levels were quantified using real-time PCR. Protein levels (B, D, F) were quantified using ELISAs. Results are expressed as means +/- SEM. *P < 0.05 ranibizumab treated cells vs. non-treated cells; **P< 0.05 aflibercept treated cells vs. non-treated cells; †P<0.05 ranibizumab treated cells vs aflibercept-treated cells.

**Fig 3:**
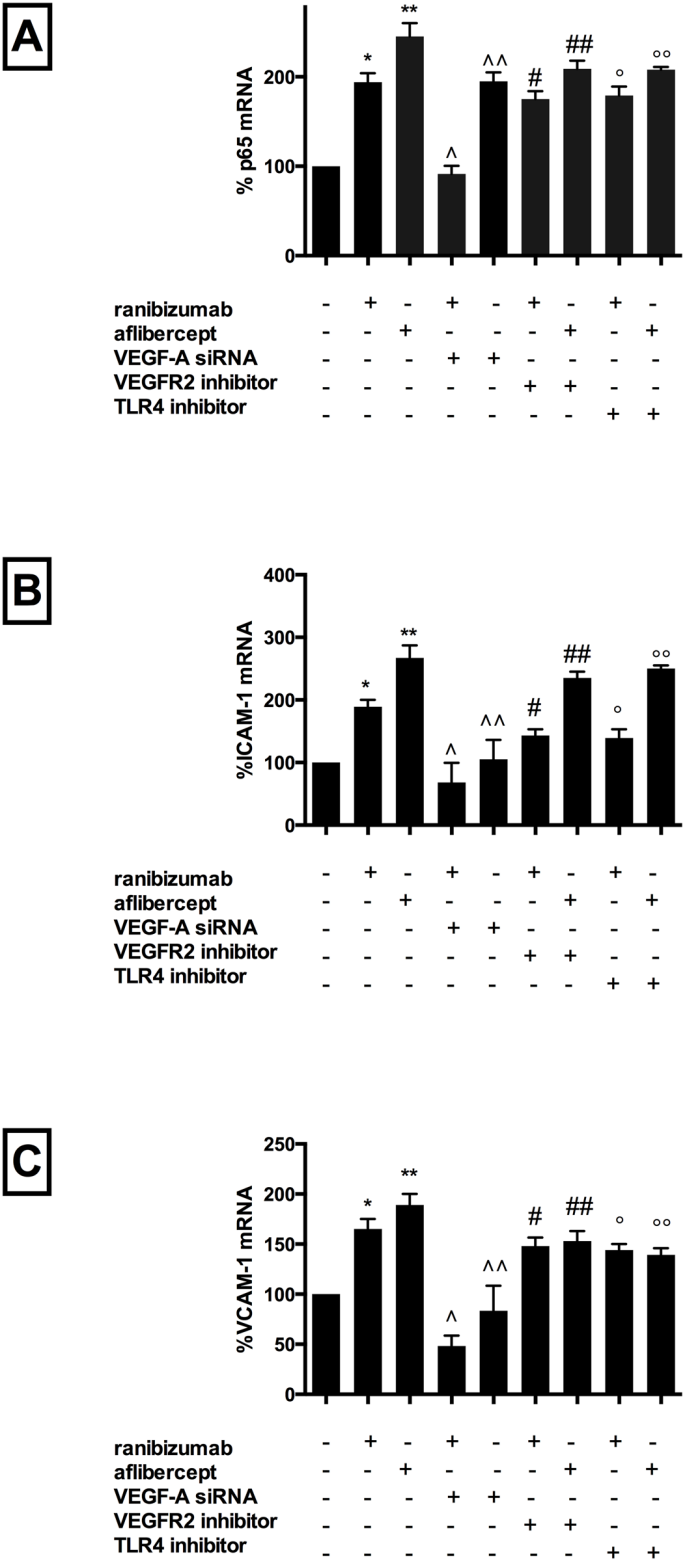
Ranibizumab and aflibercept increased p65, ICAM-1 and VCAM-1 mRNA in HCAECs, with these effects attenuated by blocking VEGF-A gene synthesis, or VEGFR2/VEGF-A ligand binding or TLR4 receptor inhibition. HCAECs were incubated with ranibizumab (0.1nM) or aflibercept (0.45nM), or PBS (control). For VEGF-A knockout studies, HCAECs were transfected with VEGF-A siRNA 72h prior to incubation. For VEGFR2 inhibitor and TLR4 inhibitor experiments, these agents were added simultaneously with the drug. Changes in p65, ICAM-1 and VCAM-1 (panels A, B and C) mRNA levels were quantified using real-time PCR. Results are expressed as means +/- SEM. *P < 0.05 ranibizumab treated cells vs. non-treated cells, **P< 0.05 aflibercept-treated cells vs. non-treated cells, ∧P<0.05 ranibizumab-treated non VEGF-A siRNA transfected cells vs. ranibizumab-treated VEGF-A siRNA transfected cells, ∧∧P<0.05 afliberept-treated non VEGF-A siRNA transfected cells vs. abflibercept-treated VEGF-A siRNA transfected cells, # P < 0.05 ranibizumab-treated cells vs. ranibizumab-treated VEGFR2 inhibitor treated cells; ##P< 0.05 aflibercept-treated cells vs. aflibercept treated VEGFR2 inhibitor treated cells, ° P < 0.05 ranibizumab treated cells vs. ranibizumab-treated TLR4 inhibitor treated cells; °°P< 0.05 aflibercept-treated cells vs. aflibercept treated TLR4 inhibitor treated cells.

Compared with untreated cells, co-incubation with either drug significantly increased mRNA expression and protein levels of CCL2 (mRNA: ranibizumab 110%, afliberept 174%; protein: ranibizumab 155%, aflibercept 255%, Fig 2, Panels A and B), CCL5 (mRNA: ranibizumab 112%, afliberept 167%; protein: ranibizumab 262%, aflibercept 322%, Fig 2, Panels C and D) and CXC3L1 (mRNA: ranibizumab 165%, afliberept 229%; protein: ranibizumab 276%, aflibercept 318%, Fig 2, Panels E and F) versus untreated cells. Gene expression for p65 (ranibizumab 94%, afliberept 145%, Fig 3, Panel A), ICAM-1 (ranibizumab 189%, afliberept 267%, Fig 3, Panel B), and VCAM-1 (ranibizumab 165%, afliberept 189%, Fig 3, Panel C) was also significantly increased versus untreated cells. (p<0.05 for all). Notably, for all inflammatory mediators, when drugs were added at equivalent Cmax concentrations (ranibizumab 0.11nM, aflibercept 0.45nM), aflibercept induced a significantly higher inflammatory response than ranibizumab (p<0.05 for all).

### 2. Pro-inflammatory effects of both drugs are attenuated by inhibiting VEGF-A but not VEGF-B synthesis

To determine whether VEGF-A or VEGF-B participate in the observed pro-inflammatory effects of the drugs, siRNAs were used to knock down these proteins in endothelial cells. Cell transfection with VEGF-A or VEGF-B siRNA alone had no effect on mRNA/protein levels of any inflammatory mediator.

**Fig 4:**
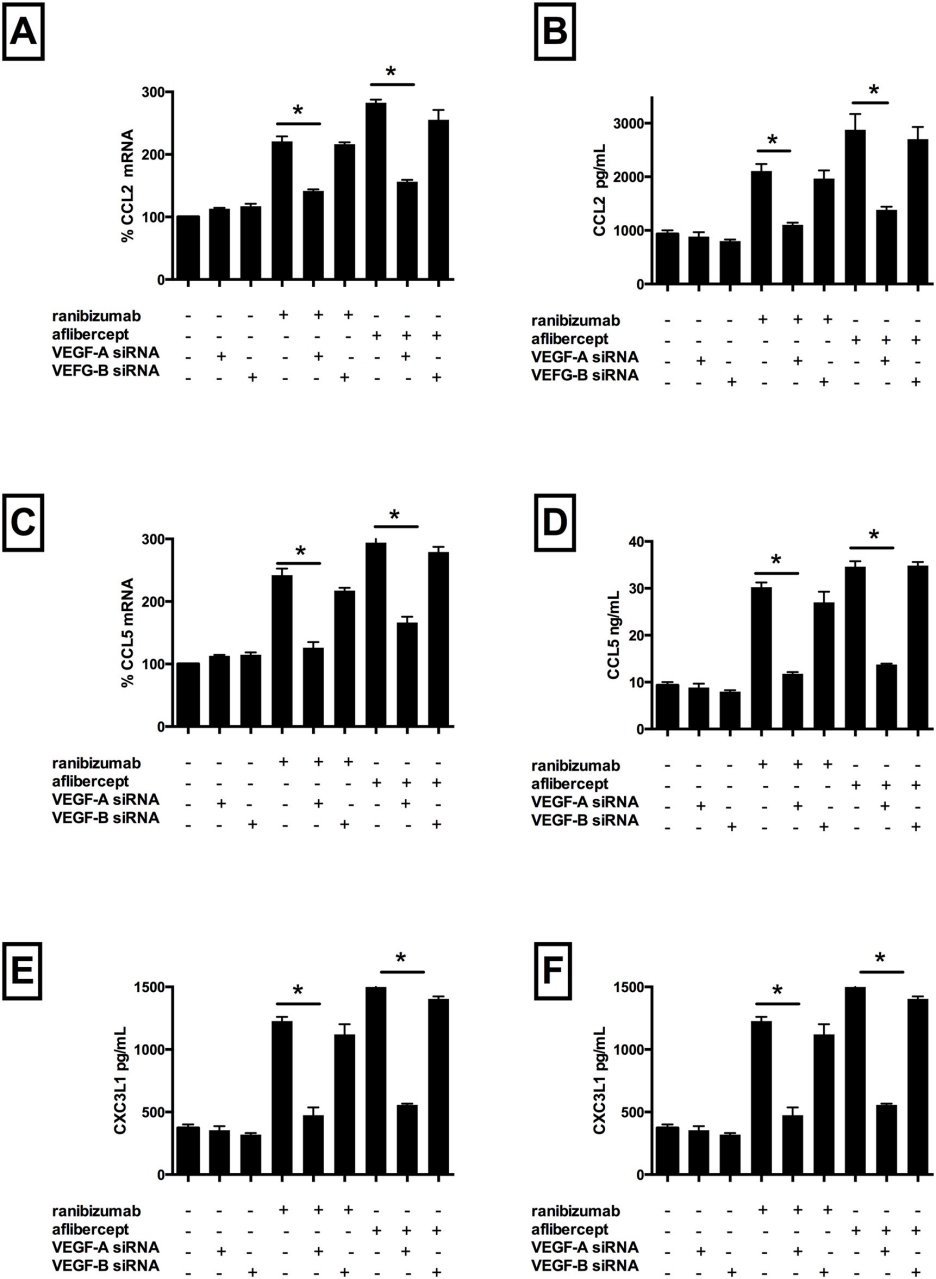
Inhibition of VEGF-A gene synthesis decreased ranibizumab and aflibercept mediated CCL2 CCL5, and CX3CL1 mRNA and protein levels in HCAECs. HCAECs were transfected with VEGF-A siRNA or VEGF-B siRNA. After 72h, cells were co-incubated with ranibizumab (0.1nM) or aflibercept (0.45nM), or PBS (control) for 16 h. Changes in CCL2, CCL5 and CXC3L1 (panels A, C and E) mRNA levels were quantified using real-time PCR. Protein levels (B, D, F) were quantified using ELISAs. Results are expressed as means +/- SEM. *P < 0.05 ranibizumab-treated non VEGF-A siRNA transfected cells vs. ranibizumab-treated VEGF-A siRNA transfected cells; **P< 0.05 aflibercept-treated non VEGF-A siRNA transfected cells vs. aflibercept-treated VEGF-A siRNA transfected cells.

Ranibizumab co-incubation with cells transfected with VEGF-A siRNA lead to a significant reduction in mRNA levels of CCL2 (by 80%) (Fig 4, Panel A), CCL5 (by 116%) (Fig 4, Panel C), CXC3L1 (by 109%) (Fig 4, Panel E), p65 (by 102%) (Fig 3, Panel A), ICAM-1 (by 126%) (Fig 3, Panel B) and VCAM-1 (by 125%) (Fig 3, Panel C) as well as protein levels of CCL2 (by 110%) (Fig 4, Panel B), CCL5 (by 193%) (Fig 4, Panel D) and CXC3L1 (by 213%) (Fig 4, Panel F) compared with non-transfected cells treated with ranibizumab (p<0.05 for all).

Aflibercept co-incubation with cells transfected with VEGF-A siRNA lead to a significant reduction in mRNA levels of CCL2 (by 126%) (Fig 4, Panel A), CCL5 (by 128%) (Fig 4, Panel C), CXC3L1 (by 166%) (Fig 4, Panel E), p65 (by 50%) (Fig 3, Panel A), ICAM-1 (by 172%,) (Fig 3, Panel B) and VCAM-1 (by 109%,) (Fig 3, Panel C) as well as protein levels of CCL2 (by 168%) (Fig 4, Panel B), CCL5 (by 218%) (Fig 4, Panel D) and CXC3L1 (by 266%) (Fig 4, Panel F) compared with non-transfected cells treated with aflibercept (p<0.05 for all). Notably, co-incubation with cells transfected with scrambled VEGF-A and either drug resulted in inflammatory mediator mRNA/protein levels comparable to drug treated non-transfected cells. Similarly, drug treated VEGF-B transfected cells expressed similar levels of inflammatory mediators compared with non-transfected drug treated cells. VEGF-A mRNA and secreted protein levels decreased significantly in VEGF-A siRNA transfected cells (but not VEGF-B siRNA transfected cells) and VEGF-A protein levels decreased further when VEGF-A siRNA transfected cells were treated with either drug. These data demonstrate, therefore, that the presence of both VEGF-A protein, but not VEGF-B, and drug is necessary to induce inflammation.

### 3. Pro-inflammatory effects of both drugs are attenuated by inhibition of VEGFR2

Drug treated cells were also co-incubated with a competitive VEGFR2 inhibitor. Incubation with the inhibitor alone had no effect on inflammatory mediator expression, however did significantly reduce VEGF-A protein levels (by 30%).

**Fig 5:**
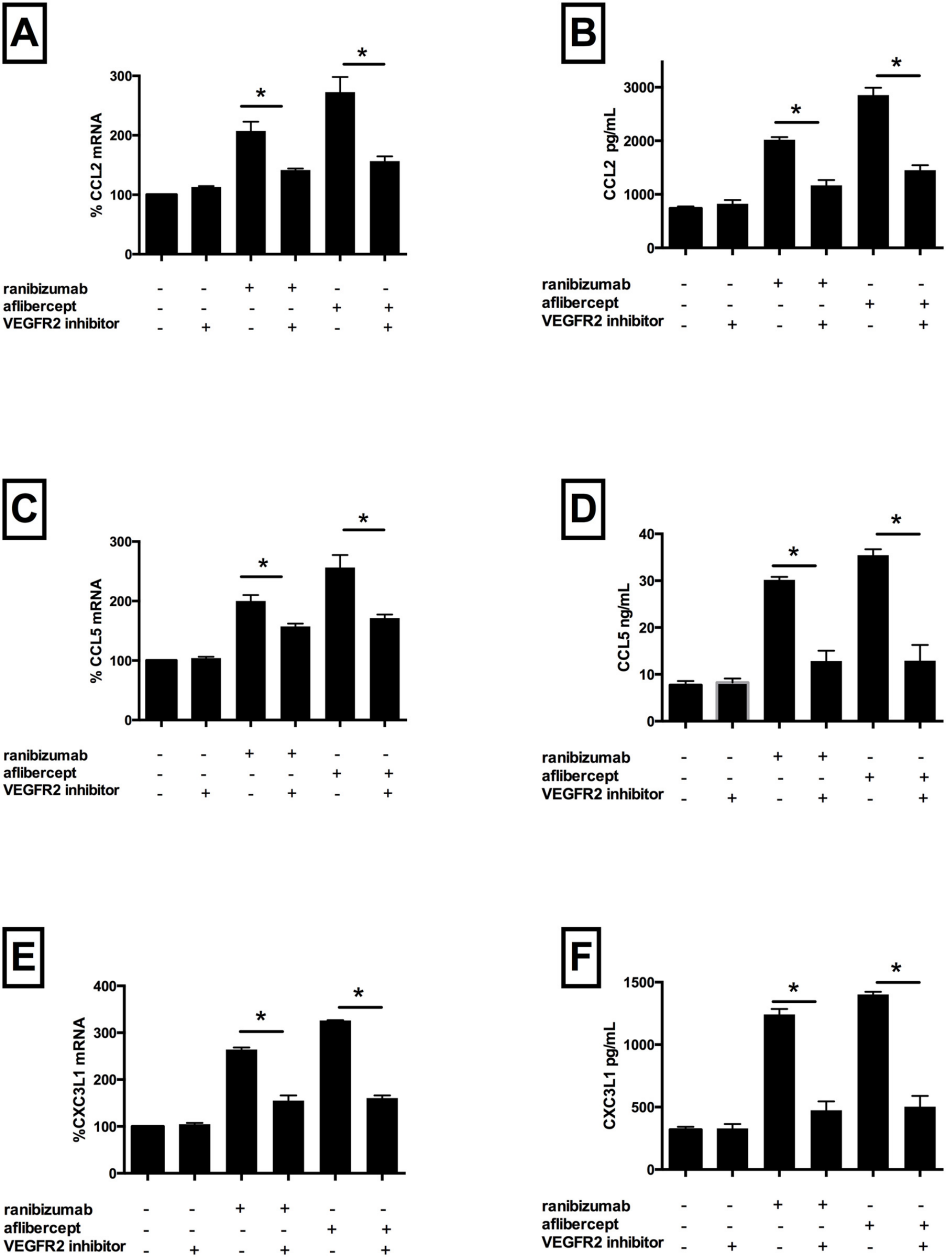
VEGFR2/VEGF-A ligand binding decreased ranibizumab and aflibercept mediated CCL2, CCL5, and CX3CL1 mRNA and protein levels in HCAECs. HCAECs were incubated with ranibizumab (0.1nM) or aflibercept (0.45nM), or PBS (control) and a VEGFR2 inhibitor for 16 h. Changes in CCL2, CCL5 and CXC3L1 (panels A, C and E) mRNA levels were quantified using real-time PCR. Protein levels (B, D, F) were quantified using ELISAs. Results are expressed as means +/- SEM. *P < 0.05 ranibizumab-treated cells vs. ranibizumab-treated VEGFR2 inhibitor treated cells; **P< 0.05 aflibercept-treated cells vs. aflibercept treated VEGFR2 inhibitor treated cells.

However, co-incubation with ranibizumab plus blocker significantly attenuated mRNA expression of CCL2 (by 66%) (Fig 5, Panel A), CCL5 (by 42%) (Fig 5, Panel C), CXC3L1 (by 95%) (Fig 5, Panel E), p65 (by 19%) (Fig 3, Panel A), ICAM-1 (by 46%) (Fig 3, Panel B) and VCAM-1 (by 16%) (Fig 3, Panel C) as well as protein expression of CCL2 (by 91%) (Fig 5, Panel B), CCL5 (by 209%) (Fig 5, Panel D) and CXC3L1 (by 222%) (Fig 5, Panel F) versus cells treated with drug alone (p<0.05 for all). Similarly, co-incubation with aflibercept plus blocker significantly attenuated mRNA expression of CCL2 (by 121%) (Fig 5, Panel A), CCL5 (by 85%) (Fig 5, Panel C), CXC3L1 (by 166%) (Fig 5, Panel E), p65 (by 36%) (Fig 3, Panel A), ICAM-1 (by 32%) (Fig 3, Panel B) and VCAM-1 (by 36%) (Fig 3, Panel C) and protein expression of CCL2 (by 150%) (Fig 5, Panel B), CCL5 (by 242%) (Fig 5, Panel D) and CXC3L1 (by 240%) (Fig 5, Panel F) versus cells treated with drug alone (p<0.05 for all). Drug treated cells co-incubated with the inhibitor further reduced VEGF-A protein levels (ranibizumab 15%, aflibercept 35% versus inhibitor alone). VEGF-A gene expression was not significantly affected by incubation with blocker alone or co-incubation with the blocker and either drug. Together, these data suggest that blocking VEGFR2 in the absence of drug did not have pro-inflammatory effects, however did lead to a reduction of secreted VEGF-A protein levels (possibly due to cross-reactivity for both the receptor and secreted protein). Addition of drug plus inhibitor significantly attenuated pro-inflammatory drug effects, likely driven by reduction of secreted VEGF-A by the inhibitor, therefore suggesting that, in similar fashion to the results seen with VEGF-A siRNA knockdown, both drug and secreted VEGF-A are necessary to induce inflammation.

### 4. Pro-inflammatory effects of both drugs are attenuated by inhibition of Toll like receptor 4 receptor

**Fig 6:**
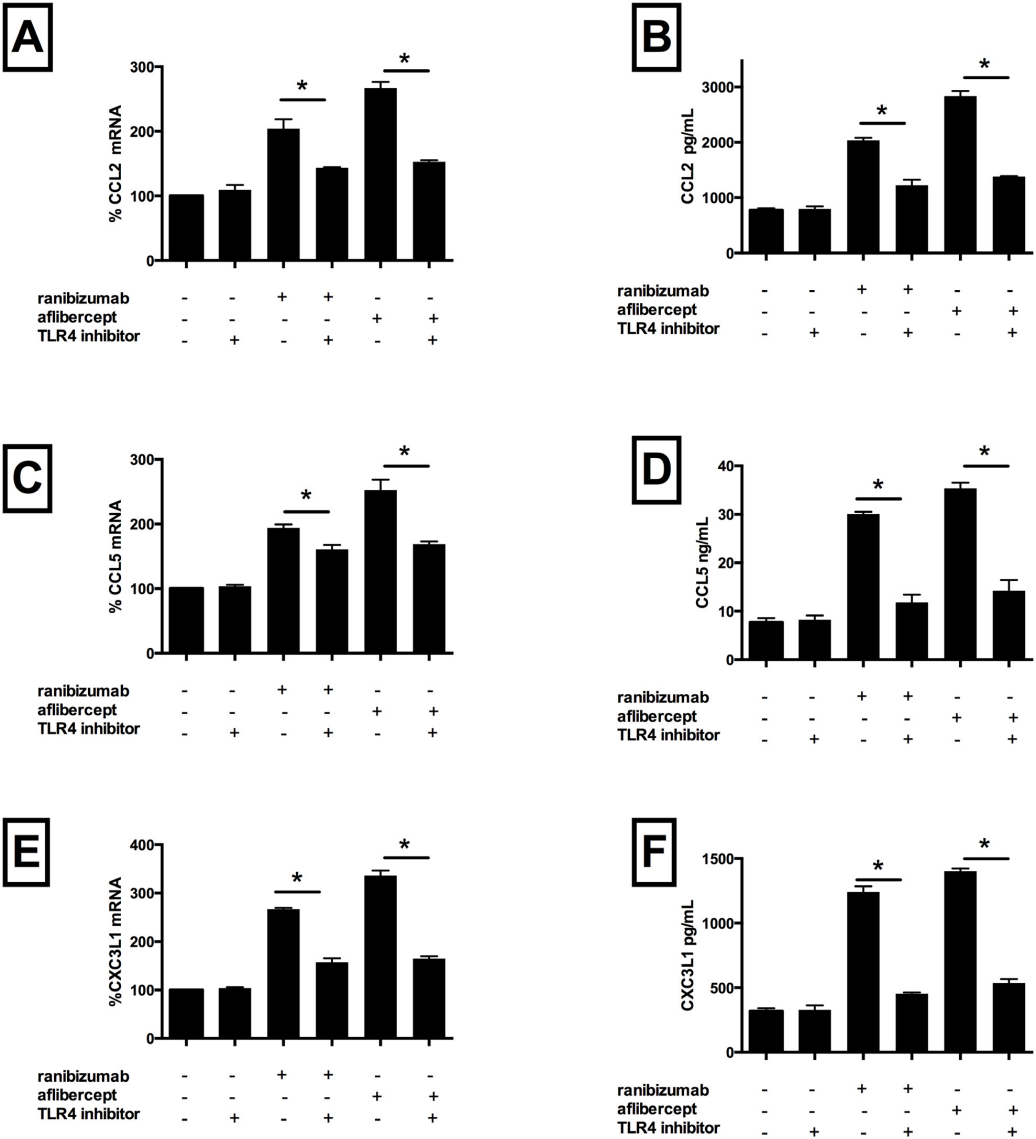
TLR4 receptor inhibition decreased ranibizumab and aflibercept mediated CCL2, CCL5, and CX3CL1 mRNA and protein levels in HCAECs. HCAECs were incubated with ranibizumab (0.1nM) or aflibercept (0.45nM), or PBS (control) and a TLR4 inhibitor for 16 h. Changes in CCL2, CCL5 and CXC3L1 (panels A, C and E) mRNA levels were quantified using real-time PCR. Protein levels (B, D, F) were quantified using ELISAs. Results are expressed as means +/- SEM. *P < 0.05 ranibizumab treated cells vs. ranibizumab-treated TLR4 inhibitor treated cells; **P< 0.05 aflibercept-treated cells vs. aflibercept treated TLR4 inhibitor treated cells.

To determine whether the pro-inflammatory effects of these drugs are mediated by activation of the TLR4 receptor, with downstream activation of the NF-kB pathway, a TLR4 receptor blocker was co-incubated with drug-treated cells Addition of the blocker alone had no effect on expression of inflammatory mediators or VEGF-A protein levels. However, co-incubation with ranibizumab plus TLR4 receptor blocker significantly reduced mRNA levels of CCL2 (by 60%) (Fig 6, Panel A), CCL5 (by 35%) (Fig 6, Panel C), CXC3L1 (by 109%) (Fig 6, Panel E), p65 (by 15%) (Fig 3, Panel A), ICAM-1 (by 50%) (Fig 3, Panel B) and VCAM-1 (by 21%) (Fig 3, Panel C) versus cells treated with ranibizumab alone (p<0.05 for all). CCL2, CCL5 and CXC3L1 protein levels were similarly significantly reduced compared with ranibizumab treatment only (92%, 209%, 220%, respectively, p<0.05 for all, Fig 6, Panels B, D, F). Similarly, addition of the TLR4 blocker to aflibercept-treated cells significantly reduced mRNA levels of CCL2 (by 115%) (Fig 6, Panel A), CCL5 (by 84%) (Fig 6, Panel C), CXC3L1 (by 172%) (Fig 6, Panel E), p65 (by 37%) (Fig 3, Panel A), ICAM-1 (by 16%) (Fig 3, Panel B) and VCAM-1 (by 49%) (Fig 3, Panel C) versus cells treated with aflibercept alone (p<0.05 for all). CCL2, CCL5 and CXC3L1 protein levels were also significantly reduced compared with aflibercept treatment only (164%, 241%, 243%, respectively, p<0.05 for all, Fig 6, Panels B, D, F). These data therefore demonstrate that these drugs mediate their inflammatory effects via activation of cell surface TLR4 receptor, with subsequent activation of intra-cellular NF-kb, thereby driving a pro-inflammatory cascade.

## Discussion

There are three key findings from our study. Firstly, both ranibizumab and aflibercept markedly increase expression of atherosclerosis associated inflammatory mediators in endothelial cells, with aflibercept being significantly more pro-inflammatory than ranibizumab at equivalent Cmax concentrations. Secondly, binding of the drug to secreted VEGF-A is crucial in promoting inflammation. Finally, inflammation appears to be mediated, at least in part, by activation of the Toll-like receptor 4 on the endothelial cell surface.

A number of clinical trials have demonstrated an association between anti-VEGF therapies and increased cardiovascular events, though these have not been seen consistently across all studies. Use of the VEGF inhibitor bevacizumab in the treatment of various cancers has been linked to an increase in arterial thromboembolic events [21] [22]. Recently, these observations have been extended to patients receiving intra-vitreal VEGF inhibitors. For example, 2 year data from the IVAN trial [23], demonstrated a statistically significant increase in risk of systemic adverse events (including cardiovascular) with bevacizumab compared with ranibizumab. Moreover, higher dose ranibizumab increased stroke incidence risk in those patients at high baseline risk [24]. Similarly, data from the VIEW studies demonstrated a significant increase in stroke incidence in patients over 85 years treated with aflibercept versus ranibizumab. Finally, diabetic patients have been shown to be more likely to have a stroke at higher dose ranibizumab than non-diabetic subjects [25]. In contrast, Campbell et al found no association between ranibizumab therapy and stroke incidence in elderly patients [26]. These findings may, in part, be explained by the systemic absorption of these agents, determined by the presence of an Fc fragment on bevacizumab and aflibercept, facilitating recycling and systemic absorption, whereas ranibizumab lacks an Fc receptor and accordingly has a shorter systemic half life. Avery et al have previously demonstrated marked reductions in plasma VEGF levels following bevacizumab and aflibercept injections, but with minimal reduction following ranibizumab injections, with corresponding systemic absorptions 70 fold higher for bevacizumab and 13 fold higher for aflibercept compared with ranibizumab [15]. Taken together, these data suggest that systemic absorption of intra-vitreal VEGF inhibitors may determine their adverse cardiovascular effects, particularly in those patients at high baseline risk. Accordingly, in our study, we compared Cmax concentrations of Ranibizumab and Aflibercept in order to simulate systemic effects of each drug on vascular endothelial cells. Consistent with the VIEW study, we found that at equivalent Cmax concentrations, Aflibercept was significantly more pro-inflammatory than Ranibizumab, suggesting that these effects are principally driven by systemic absorption. However as AUC provides a more accurate reflection of systemic absorption, we may have underestimated differences between two drugs.

Previously documented adverse cardiovascular side effects of VEGF inhibition include hypertension, thought due to inhibition of nitric oxide production, leading to endothelial dysfunction and vasoconstriction [27] and arterial thromboembolism via an increase in endothelial cell apoptosis, with disruption of the endothelial lining and subsequent exposure to underlying pro-coagulant factors [28] [20]. Recently, Winnik et al demonstrated that VEGF inhibition lead to an imbalance in endothelial superoxide and nitric oxide production, disrupting endothelial homeostasis and accelerating pre-existing atherosclerosis [29]. Notably, these effects are seen with both VEGF ligand blockage and/or VEGF receptor inhibition.

**Fig 7:**
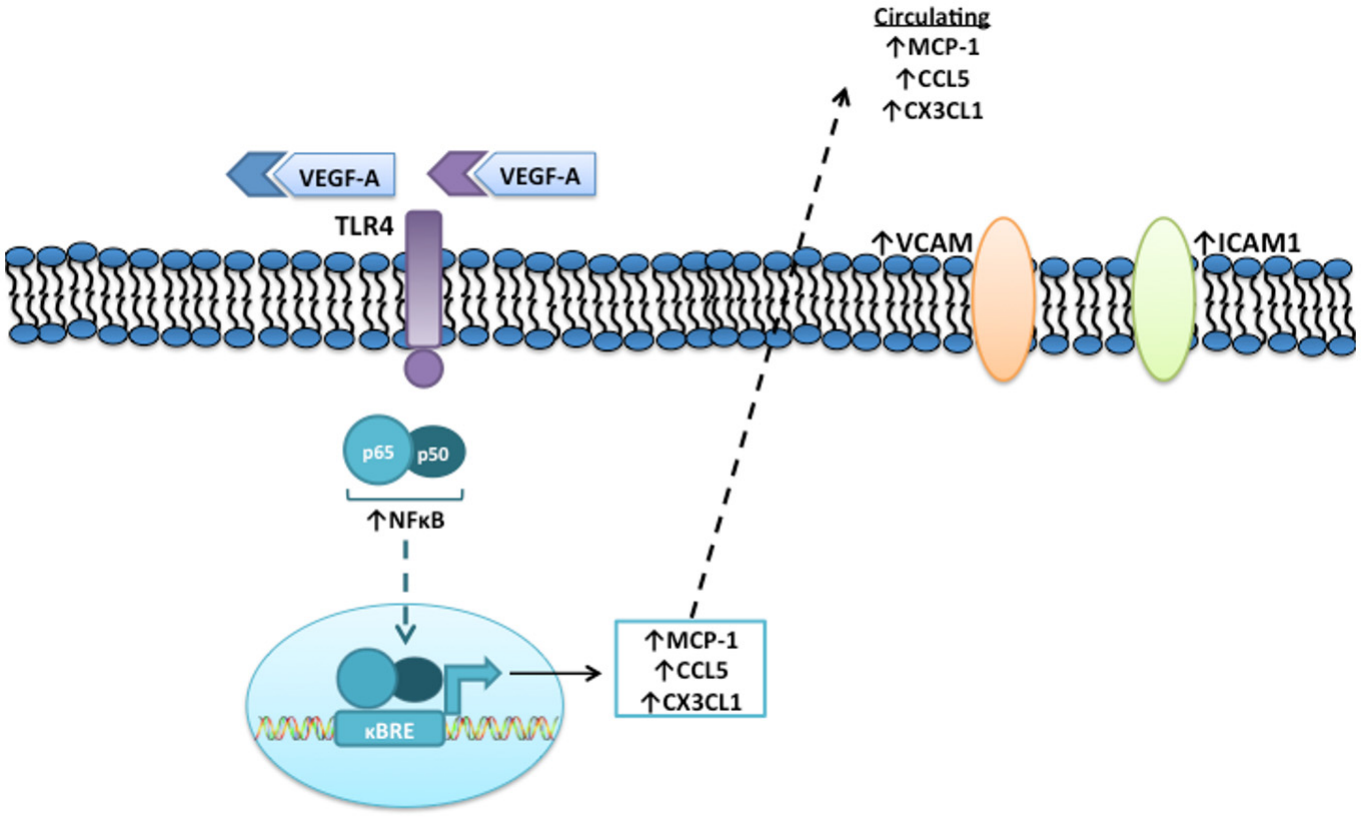
Proposed mechanism for Ranibizumab/Aflibercept induced inflammation. Binding of VEGF-A to Ranibizumab (R) or Aflibercept (A) is essential for Toll-like receptor 4 activation, with downstream activation of intra-cellular NF-κB, chemokine gene expression and protein secretion and cell adhesion molecule expression.

In contrast, the pro-inflammatory effects observed in our study are not mediated through simply through attenuation of the VEGF-A pathway, as evidenced by a lack of effect with blockade of VEGF-A gene synthesis or VEGFR2/VEGF-A inhibition in the absence of drug. We demonstrate here a novel pro-inflammatory mechanism where drug binding to secreted VEGF-A is vital to promote inflammation. This was shown either by reducing VEGF-A mRNA synthesis and therefore protein secretion or neutralization of secreted VEGF-A as a byproduct of VEGFR2 inhibition (likely due to cross-reactivity between VEGFR2 and secreted VEGF-A), both of which attenuated the inflammatory response. Further, we demonstrated that inflammation is driven, at least in part, by the TLR4 receptor on the endothelial cell surface (Fig 7).

Toll like receptor 4 has been strongly implicated in driving atherosclerosis-associated inflammation [30]. This trans-membrane receptor is widely expressed on multiple cell types that participate in atherogenesis including endothelial cells and monocytes and has been shown to bind several potentially pro-atherogenic ligands, including fibronectin-EDA, oxidized LDL, Heat Shock Protein and LPS [31]. Stimulation of TLR4 leads to activation of the NF-κB family of transcription factors, a key upstream regulator of atherosclerosis-associated inflammation. Amongst the different types of NF-κB dimers, the P65/P50 heterodimer [32], is the most common form and plays a key role in promoting expression of the chemokines CCL2, CCL5 [33], and CX3CL1 [34] as well as the cell adhesion molecules VCAM-1 and ICAM-1, all of which play critical roles in mediating inflammation, monocyte recruitment, and progresssion of atherosclerosis [35]. Also, over-expression of p65 NF-κB subunit has been demonstrated in animal models of atherosclerosis as well human atherosclerotic tissue [36]. Consistent with this athero-inflammatory cascade, in our study, both drugs lead to TLR4 receptor activation, thereby significantly increasing p65, chemokine and cell adhesion molecule expression.

Limitations of this study center around these data being limited to *in vitro* observations only. Also, aflibercept is also known to inhibit VEGF-B and PIGF, and although VEGF-B gene knockdown had no effect on inflammation, we cannot exclude effects on PIGF inhibition also driving inflammation. Moreover, we compared ranibizumab and aflibercept at each drug’s Cmax, whereas *in vivo* studies comparing intermittent administration of these drugs would be a more accurate assessment of their effects. Finally, we have not elucidated how the VEGF-A/drug complex binds and activates the TLR-4 receptor. Nevertheless, we report here, for the first time, potent pro-inflammatory effects of these drugs, in parallel with their systemic absorption, which may explain, at least in part, adverse cardiovascular events observed in clinical studies, suggesting that these agents should be used with caution in patients with high baseline cardiovascular risk.
